# Supplementary material for: Design and flight results of the VHF/UHF communication system of Longjiang lunar microsatellites
Source: Nat Commun. 2020 Jul 9;11:3425. doi: 10.1038/s41467-020-17272-8 (PMC7347839; doi:10.1038/s41467-020-17272-8)
Supplement: Supplementary file 1 — Supplementary Information [file 41467_2020_17272_MOESM1_ESM.pdf]

Supplementary Information

Design and Flight Results of the VHF/UHF Communication System of Longjiang Lunar Microsatellites

Wei et al.

Supplementary Table 1: Link budgets for UHF downlink. For a reliable link, the maximum distance between the Earth and the Moon, the largest antenna pointing loss and polarization loss and a larger target link budget were taken into account. It shows the requirements if we want to communicate with the satellite at any time and when the satellite is at any attitude. For a lucky case, the average distance between the Earth and the Moon, a smaller antenna pointing loss and polarization loss and a smaller target link budget were taken into account. It shows the minimum requirements to communicate with the satellite, if lucky enough.

Item	Unit	Reliable link	Lucky case
Range	km	400000	380000
Frequency	MHz	436	436
Transmitter output power	dBm	33	33
Transmitter antenna gain	dB	-6	-1
Free space path loss	dB	197.3	196.8
Polarization loss	dB	3	1
Other losses	dB	1	1
Receiver antenna gain	dB	30	20
Receiver input level	dBm	-144.3	-146.8
System noise figure	dB	2	2
Receiver C/N0	dB	27.7	25.2
C/N0 threshold	dB	25	25
Link margin	dB	2.7	0.2

Supplementary Table 2: Link budgets for VHF uplink. For a reliable link, the maximum distance between the Earth and the Moon, the largest antenna pointing loss and polarization loss and a larger target link budget were taken into account. It shows the requirements if we want to communicate with the satellite at any time and when the satellite is at any attitude. For a lucky case, the average distance between the Earth and the Moon, a smaller antenna pointing loss and polarization loss and a smaller target link budget were taken account. It shows the minimum requirements to communicate with the satellite, if lucky enough.

Item	Unit	Reliable link	Lucky case
Range	km	400000	380000
Frequency	MHz	145.9	145.9
Transmitter output power	dBm	60	53
Transmitter antenna gain	dB	21	18
Free space path loss	dB	187.8	187.3
Polarization loss	dB	3	1
Other losses	dB	1	1
Receiver antenna gain	dB	-15	-11
Receiver input level	dBm	-125.8	-129.3
Receiver Sensitivity	dBm	-132	-132
Link Margin	dB	6.2	2.7

Supplementary Table 3: Specifications of Longjiang-1/2 VHF/UHF radio.

Specification	Value
Frequency range	435 ~ 438 MHz (downlink)<br>145.8 ~ 146 MHz (uplink)
Downlink modulation	GMSK, $BT = 0.5$ (telemetry)<br>4FSK (beacon)
Downlink symbol rate	250/500 bps (telemetry)<br>4.375 bps (beacon)
Downlink channel coding	Turbo code, $r = 1/2$, 1/4 or 1/6 (telemetry)<br>$r = 1/2$, $k = 32$ convolutional code (beacon)
Transmitter output power	2 W
Uplink modulation	GMSK (telecommand)<br>2FSK with pseudo-random GMSK symbol shaping<br>(low rate telecommand)
Uplink symbol rate	250 bps (telecommand)<br>7.8125 bps (low rate telecommand)
Uplink channel coding	(64, 32) Reed-Solomon code
Receiver sensitivity	-132 dBm (telecommand)<br>-140 dBm (low rate telecommand)
Data interface	RS422
Power interface	24.5 ~ 29.4 V
Power consumption	4.2 W (2 × RX)<br>10 W (2 × RX + 1 × TX)<br>15.8 W (2 × RX + 2 × TX)
Mass	360 g

Supplementary Table 4: Information of ground stations participating in the 5 July 2019 observations.

Station	Coordination	Type	Polarization	Gain
Dwingeloo	52.83 N, 6.35 E	Dish, 25 m aperture	Vertical / Horizontal	36 dBi
Wakayama	34.27 N, 135.15 E	Dish, 12 m aperture	Horizontal	31 dBi
Shahe	40.12 N, 116.23 E	Dish, 12 m aperture	Circular, left hand	30 dBi
Harbin	45.95 N, 126.80 E	Yagi array, 8×15 elements	Horizontal	24 dBi

Supplementary Table 5: Orbital elements determined by CDSN for comparison with UHF VLBI.

Item	Unit	Value
Coordination system	-	Moon ICRF
Orbit epoch	-	8 Jun 2018 23:00:00 UTCG
Semi-major axis	km	8749.7953
Eccentricity	-	0.736543883
Inclination	°	40.03274077
Right ascension of ascending node	°	320.9917409
Argument of periapsis	°	97.61703801
Mean anomaly	°	306.6934452
